# Semantic Dementia Shows both Storage and Access Disorders of Semantic Memory

**DOI:** 10.1155/2014/508960

**Published:** 2014-03-09

**Authors:** Yumi Takahashi, Kenichi Meguro, Masahiro Nakatsuka, Mari Kasai, Kyoko Akanuma, Satoshi Yamaguchi

**Affiliations:** Department of Geriatric Behavioral Neurology, Tohoku University Graduate School of Medicine, 2-1, Seiryo-machi, 980-8575 Sendai, Japan

## Abstract

*Objective*. Previous studies have shown that some patients with semantic dementia (SD) have memory storage disorders, while others have access disorders. Here, we report three SD cases with both disorders. *Methods*. Ten pictures and ten words were prepared as visual stimuli to determine if the patients could correctly answer names and select pictures after hearing the names of items (Card Presentation Task, assessing memory storage disorder). In a second task, the viewing time was set at 20 or 300 msec (Momentary Presentation Task, evaluating memory access disorder) using items for which correct answers were given in the first task. The results were compared with those for 6 patients with Alzheimer's disease (AD). *Results*. The SD patients had lower scores than the AD group for both tasks, suggesting both storage and access disorders. The AD group had almost perfect scores on the Card Presentation Task but showed impairment on the Momentary Presentation Task, although to a lesser extent than the SD cases. *Conclusions*. These results suggest that SD patients have both storage and access disorders and have more severe access disorder than patients with AD.

## 1. Introduction

Semantic dementia (SD) is a progressive degenerative disease in which phonology and syntax of speech are retained, but semantic memory of speech is selectively impaired [1–3]. Semantic memory is a subclass of long-term memory that was introduced into cognitive psychology as a concept in contrast to episodic memory [4]. Thus, semantic memory represents memory of socially shared knowledge and concepts independent from personal experience and temporal context. Semantic memory disorders include “storage disorder,” in which stored information is lost [5], and “access disorder,” in which access to stored memory is impaired [6].

In Alzheimer's disease (AD), semantic memory disorder is not required for clinical diagnosis but often appears in the disease course [5, 7, 8]. In previous studies of semantic memory disorder in SD and AD, Bozeat et al. [9] observed consistent errors in drawings and words in a semantic matching task performed by 10 patients with SD, thus showing the presence of semantic storage disorder. Yoshino and Kato [10] suggested the possibility of access disorder in SD in a case report of a patient with SD with impairments that differed between naming of drawings and comprehension of words. It was also proposed that localized injury in the left temporal lobe is involved in storage disorder, whereas degenerative changes, rather than localized injury, in the bilateral temporal lobes may be involved in access disorder. These findings indicate the inconsistency in the results of previous studies. Moreover, the presence of both storage and access disorders of semantic memory has not been demonstrated in patients with SD, and the relationship between impairments of storage of and access to semantic memory has not been investigated.

We encountered three patients with SD who appeared to have both storage and access disorders of semantic memory. The objective of this study was to examine the relationship between these two disorders in these patients by investigating differences in the level of semantic memory based on a presentation pattern (images or words) and the duration of presentation using tasks for which the semantic memory appeared to have been retained.

## 2. Subjects

### 2.1. Diagnosis and Characteristics

The three patients were all diagnosed with SD based on the diagnostic criteria established by Neary et al. [2]. The patients were 59, 69, and 84 years old and all were right-handed women. Their chief complaints were word finding difficulties and amnesia. They had no relevant personal or familial medical histories. For two or three years, they had frequently became anxious about financial problems with their families, communication with others had decreased, and words frequently did not come out when they were asked questions. They stopped cooking and had difficulties with activities of daily living (ADL), such as use of a washing machine and television remote control. They were unable to calculate money, and going out and shopping alone became difficult. At their first visits, there were no particular general internal medicine or test findings. Neurological findings also showed no particular abnormality.

The clinical characteristics of the three patients are shown in Table 1. The disease stages were 4-5 (moderate to slightly severe reduction of cognitive function) on the Functional Assessment Stage of Alzheimer's Disease (FAST) scale, which is used to evaluate the severity of cognitive function based on observation of ADL. Magnetic resonance imaging (MRI) showed no cerebral infarction, but mild cerebral atrophy was noted mainly in the left temporal lobe in the coronal view. Executive function assessed by the Trail Making Test A and word fluency task and language function examined by the Western Aphasia Battery are shown in Table 2.

### 2.2. Control Subjects

The control subjects were 6 patients (2 men and 4 women) with AD. The patients were diagnosed with probable AD based on the NINCDS-ADRDA criteria and had similar FAST scores to those of the three SD cases. The mean (std. dev.) age, years of education, and MMSE score were 78.0 (5.6), 8.7 (0.5), and 17.5 (2.4), respectively.

## 3. Semantic Memory Task 1: Tasks Based on Card Presentation

### 3.1. Naming

First, to investigate storage disorder of semantic memory, a task based on card presentation using drawings was performed. Ten cards used for the picture classification task in the standard higher visual perception test were used. The cards were individually presented and the patient was asked to name the pictures.

### 3.2. Coupling Task

Ten words and 10 drawing cards were used in this task. Five cards for selection, including the target card, were presented simultaneously with a stimulation card, and the patient selected a semantically related card. For example, the target card “bat” should be selected for the stimulation card “ball.” The arrangement of cards was changed in each trial.

### 3.3. Matching Task

The 10 words and 10 drawing cards described above were also used in this task. For drawing-to-word matching, a targeting word was selected among 4 words so as to match each drawing, with the opposite approach used for word-to drawing matching.

### 3.4. Results for Semantic Memory Task 1

The results of the card presentation tasks for the SD and AD patients are shown in Table 3. All AD patients succeeded in coupling of drawings to words. Three patients failed with naming of a single drawing but could correctly answer on cue of the initial sound. One patient wrongly named “match” as a semantically similar word, “lighter,” but could correctly answer in the second trial after being asked “it is a lighter, is not it?” The correct answer rate for each task was higher in the AD patients than in the SD patients, and the AD patients made no errors common to the drawing, suggesting that semantic memory was retained.

SD patient number 1 could not name the drawings. She was able to make semantic couples for 4 drawing cards and to match 3 drawings to the target words and 2 words to the target drawings. The other two SD patients had higher scores than those for patient number 1, but lower scores compared to the AD patients.

Based on these findings, the inability of the SD patients to perform all tasks was considered to be due to storage disorder of semantic memory. However, even for the cards for which they could make couples, the results were inconsistent between the words and pictures, suggesting that semantic memory was stored but that the access pathway to the memory was impaired. If access disorder is present, this result should decline further when the duration of presentation of the task is shortened and rise when it is prolonged. Thus, in Task 2, we presented the cards to the patients using a shorter duration.

## 4. Semantic Memory Task 2: Task with Momentary Presentation

### 4.1. Method

The pictures used in the picture classification task in the standard higher visual perception test present only inanimate objects. Therefore, we prepared 10 photographs including those of natural items to avoid a categorical bias. To unify the speech processing speed, we selected 10 natural and 10 inanimate objects that can be expressed with 3 hiragana letters and selected 5 items for which the patient was able to do two-way matching: photograph-to-word and word-to-photograph matching. The tasks were prepared using computer software (MATLAB, Matrix Laboratory) and displayed for 20 or 300 msec on a 14-inch CRT monitor (Cathode Ray Tube). The letter size was 1.3 × 1.3 cm.

Regarding the understanding of instructions in momentary presentation and visual recognition of the display, a single hiragana letter was presented for 20 msec and the patient read it aloud. The abilities of the patients to distinguish shapes and to read aloud and their understanding of the test procedure were confirmed before the test was performed. We did not compare hiragana and Chinese characters, since the latter is difficult to test in patients with dementia.

To complete the photograph task, the patient named the photograph (naming) and pointed at one card indicating the photograph out of 4 hiragana word cards (pointing). For the word task, the patient read the word aloud and pointed at one photograph identifying the word out of 4 photograph cards.

### 4.2. Results of Semantic Memory Task 2

The correct answer rates for naming photographs presented for 300 and 20 msec for the SD patients and the AD group (Momentary Presentation Task, Pictures) are shown in Table 3. Both groups showed a decrease in these rates with shortening of the presentation time, with a particularly marked decrease in the SD patients. The correct answer rates for reading aloud hiragana words presented for 300 and 20 msec are also shown (Momentary Presentation Task, Words). There were relatively small differences in these rates between the SD patients and the AD group.

## 5. Discussion

The three SD patients showed common errors in tasks of naming and semantic matching of specific items, indicating storage disorder of semantic memory. However, they retained semantic memory for other items. Presentation of items without storage disorder for a short period caused a marked reduction in correct answers, suggesting the additional presence of access disorder. Correct answers were also reduced by shortening the duration of presentation in the AD group, but the effect was smaller than that in the SD patients. Based on the tasks used in this study, no apparent storage disorder was observed in the AD group.

Our results suggest that storage disorder of semantic memory was present in the SD patients, compared to an AD group with a similar level of ADL disability. In addition, the presence of access disorder was suggested for items with retained semantic memory. These findings may support the theory of storage disorder in SD proposed by Bozeat and the theory of access disorder in SD proposed by Yoshino et al. Our measurement of the severity of access disorder and comparison with that in AD suggest that impairments of access to semantic memory in terms of visual representation (pictures and photographs) and linguistic representation (hiragana words) were severe in our patients.

The scores for the two tasks were case 1 > case 3 > case 2 for task 1 (storage disorder) and case 1 > case 3 = case 2 for task 2 (access disorder), and the results suggest coexistence of access and storage disorders. However, we cannot exclude the possibility that access disorder might also be induced by executive dysfunction in SD, which might be more important than that in AD. Cerebral atrophy, mainly in the left temporal lobe, was observed in the SD patients, suggesting that the pattern of disorder differs due to laterality of neurological lesions in the brain, as shown by Yoshino et al. Further accumulation of cases is needed to clarify the developmental mechanism of semantic memory disorder in SD, which may include progression of access disorder to storage disorder.

Finally, we note two points that need to be considered in future studies. First, differences between hiragana and kanji characters should be examined. Kanji characters are more complex than hiragana and the control of visual complexity between two scripts remains to be studied. Second, the difficulty of performing the tasks should be controlled to exclude the possibility that the 20 msec test was just more difficult to perform compared to the 300 msec test.

## Figures and Tables

**Table 1:** Clinical characteristics of three SD cases.

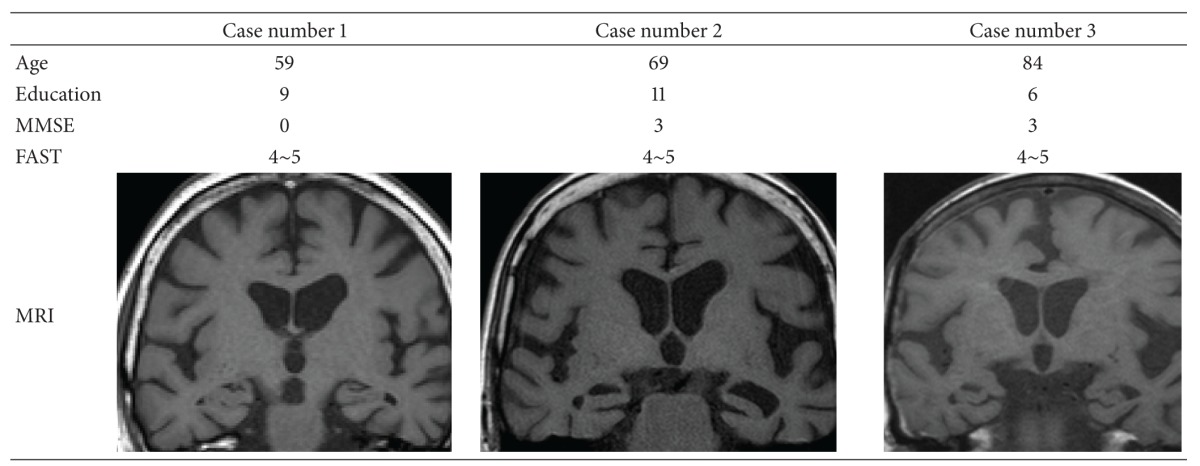

SD: semantic dementia; MMSE: mini mental state exam; FAST: functional assessment staging of Alzheimer's disease; MRI: magnetic resonance imaging.

**Table 2:** Neuropsychological findings of three SD cases.

	Case number 1	Case number 2	Case number 3
TMT-A	ND	ND	221 sec
Word fluency	0	1	3
WAB			
Auditory comprehension (60)	41	47	57
Naming (60)	1	33	51
Repetition (100)	22	16	68

SD: semantic dementia; TMT-A: trail making test-A; WAB: Western Aphasia Battery.

**Table 3:** Results on semantic memory tasks.

	Case number 1	Case number 2	Case number 3	AD

Card presentation task				
Drawings				
Naming (10)	0	2	5	9
Combination (10)	4	6	6	10
Matching				
Drawings to words (10)	3	6	7	10
Words to drawings (10)	2	6	9	10
Momentary presentation task				
300 ms				
Pictures (100)	30	80	80	93
Words (100)	60	90	80	100
20 ms				
Pictures (100)	20	40	30	57
Words (100)	80	60	60	71

SD: semantic dementia; AD: Alzheimer's disease.
